# Expression Profile and Ligand Screening of a Putative Odorant-Binding Protein, AcerOBP6, from the Asian Honeybee

**DOI:** 10.3390/insects12110955

**Published:** 2021-10-20

**Authors:** Huiting Zhao, Zhu Peng, Li Huang, Shuguo Zhao, Miaomiao Liu

**Affiliations:** College of Life Science, Shanxi Agricultural University, Jinzhong 030801, China; pzhu66@126.com (Z.P.); a2604775909@163.com (L.H.); zhaoshuguo019@163.com (S.Z.); byh806241@163.com (M.L.)

**Keywords:** honeybee, odorant binding protein, fluorescence binding assay, EAG

## Abstract

**Simple Summary:**

The olfactory sensillum, which is located in the antenna of insects, is the basic unit of the olfactory organ. Olfactory-related genes are expressed in the sensillum. It is believed that the process of olfaction recognition is mainly mediated by two gene families, odorant binding proteins (OBPs) and olfactory receptors (ORs). The honeybee possesses a large numbers of ORs, but few OBPs. Up to now, the function of OBPs in the honeybee has not yet been fully elucidated. In order to reveal the specific role of OBPs from *Apis cerana cerana*, we selected an OBP gene, AcerOBP6, which is highly expressed in the antennae of worker bees, acquired a purified protein via a prokaryotic expression system, and analyzed its function using bioinformatics, molecular biology, and electrophysiology. According to the result, AcerOBP6 was a protein with extensive binding affinity, and we speculated that its function was chiefly related to foraging. Overall, this research not only explains the essential role of OBPs in ligand binding, but also provides valuable resources to help researchers further understand the nature and mechanism of the olfactory system.

**Abstract:**

Olfaction is essential in some behaviors of honeybee, such as nursing, foraging, attracting a mate, social communication, and kin recognition. OBPs (odorant binding proteins) play a key role in the first step of olfactory perception. Here, we focused on a classic OBP with a PBP-GOBP domain from the Asian honeybee, *Apis cerana cerana*. Beyond that, the mRNA expression profiles and the binding affinity of AcerOBP6 were researched. According to qRT-PCR analysis, AcerOBP6 transcripts were mainly expressed in the antennae of forager bees. In addition, we found that the expression level of AcerOBP6 was higher than that of AmelOBP6. The fluorescence competitive binding assay indicated that the AcerOBP6 protein had binding affinity with most of the tested odors, including queen pheromone, worker pheromone, and floral volatiles, among which the strongest one was linolenic acid (with a Ki value of 1.67). However, AcerOBP6 was not sensitive to the brood pheromones. A further study based on EAG assay revealed that the antennae had the strongest response to 2-heptanone. The EAG recording values of the selected ligands were all reduced after AcerOBP6 was silenced, with 8 of 14 declining significantly (*p* < 0.01) given that these odors could specifically bind to AcerOBP6. As revealed in our current study, AcerOBP6 might be a crucial protein involved in olfactory recognition for foraging. Overall, the research provides a foundation for exploring the olfactory mechanism of *A. cerana cerana*.

## 1. Introduction

Olfaction plays a significant role in the survival and reproduction of insects by modulating several of their biological behaviors. Additionally, the antennae are the main olfactory organs, with a large number of olfactory sensilla located on them [1,2,3]. OBPs, as the soluble carrier proteins with a small molecular weight (~130–150 amino acids), are secreted in the sensillum lymph of the antenna [4], and function as the first filters for odor recognition and interaction. When binding the environmental chemical molecules, OBPs deliver them to ORs (olfactory receptors) to trigger olfactory behavior [4,5]. In addition, the OBPs of insects and vertebrates are exceptionally stable to thermal and solvent denaturation [6], which makes it possible to obtain reliable proteins whose physiological functions can be researched in vitro.

The honeybee is a eusocial insect that relies on complex olfactory language in several aspects of its life. Two sister species, *Apis mellifera* and *Apis cerana*, are the major honeybees raised in China. Compared with *A. mellifera*, *A. cerana* possesses a more sensitive olfactory system, meaning that it has a good foraging ability for the use of sporadic nectar and pollen sources [7,8]. Moreover, a total of 21 putative OBPs had been identified in the *A. mellifera* genome by bioinformatic approaches [9]; 17 of these genes were found in *A. cerana cerana* [10].

Over the past few years, researches on the function of OBPs in honeybees have been reported constantly. For instance, it was found that Amel/AcerOBP1 is apt to bind with the queen pheromone [11,12,13,14], while Amel/AcerOBP2 is more liable to bind with general odors [15,16,17]. AmelOBP13 showed a good binding specificity to oleic acid and some other compounds with similar structures. AmelOBP14 is better tuned to monoterpenoid structures, and AmelOBP21 will bind the main components of the queen mandibular pheromone as well as farnesol [18]. Apart from that, AcerOBP11 could bind to bee pheromones and some floral volatiles [19]. According to a recent study, AcerOBP10 is involved in oxidative stress defense [20], and myrcene may be a ligand of AcerOBP15 [21].

In a previous study, we have identified OPBs from the antennae of *A. cerana cerana* using the RNA-Seq method. Although the AcerOBP6 transcript has a relatively high expression value, its role in olfactory coding remains unclear. In the present study, the full-length cDNA sequence of AcerOBP6 was cloned and the gene sequence characteristics and expression profiles were analyzed. Furthermore, fluorescence competitive binding, RNA interference (RNAi), and EAG assays have been used to analyze the odor binding affinity of OBP6. This research will confirm the essential role of OBPs in ligand binding, provide valuable resources for further molecular studies on the olfactory recognition mechanism of honeybees, and offer a target gene for the guidance of effective pollination.

## 2. Materials and Methods

### 2.1. Insect Rearing and Sample Preparation

Bees were reared in an apiary laboratory of Shanxi Agricultural University, Shanxi Province, China. Combs with old pupae were taken from healthy colonies and maintained in an artificial climate incubator for eclosion (75–80% relative humidity [RH]; 34 ± 0.4 °C). The newly emerged bees were marked and returned to three colonies. The antennae of worker bees from *A. cerana cerana* at 1, 5, 10, 15, 20, 25, and 30 days together with different tissues from the head, thorax, abdomen, legs, and wings of forager bees were collected separately. At the same time, the antennae of forager bees from *A. mellifera ligustica* and matured drones of *A. cerana cerana* were also collected. All the samples were immersed in liquid nitrogen immediately after collection and then stored at −80 °C until use.

### 2.2. RNA Isolation and Quantitative Real-Time PCR

Total RNA was isolated using TRIzol reagent (Invitrogen, Carlsbad, CA, USA) and the cDNA was synthesized using the PrimeScript II 1st Strand cDNA Synthesis Kit (Takara, Dalian, China), according to the manufacturer’s instructions. Apart from that, *quantitative real-time PCR* (qRT-PCR) was performed using an ABI 7500 Real-Time PCR System (Applied Biosystems, Carlsbad, CA, USA). The *AcerArp1* (GenBank No. HM640276.1) was used as the housekeeping gene. The primers used for amplification, *AcerArp1* and AcerOBP6, are shown in Table S1. Furthermore, the reaction was performed using SYBR^®^ Premix Ex Taq™ II (Tli RNaseH Plus) (TaKaRa, Dalian, China). We used a 20 μL of mixture containing 10 μL of SYBR^®^ Premix Ex Taq™ II (2×), 0.4 μL of ROX Reference Dye II (2×), 2 μL of cDNA template, 0.8 μL forward primer, 0.8 μL of reversed primer, and 6 μL of sterile water. The cycling conditions were as follows: denaturation at 95 °C for 20 s, followed by 40 cycles at 95 °C for 15 s and at 60 °C for 20 s, with melting curve analyses at 95 °C for 20 s, 60 °C for 30 s, and 95 °C for 30 s. In order to ensure data reproducibility and reliability, each sample was analyzed in biological triplicate. The relative gene expression value was calculated using the comparative 2^−ΔΔCt^ method [22].

### 2.3. Cloning Full-Length of cDNA and Sequence Analysis

The complete ORF sequence of AcerOBP6 was amplified using specific primers (Table S1), preceded by restriction enzyme sites for *BamH* I and *Hind* III. The PCR product was purified and sub-cloned into a pGEM-T vector (Tiangen Biotech, Beijing, China). After transforming the ligation product into DH5α *E. coli* cells, the positive colonies were identified by white/blue screening. Then, the positive plasmids were extracted and the sequence was confirmed by Sangon Biotech (Shanghai, China).

The amino acid sequence of AcerOBP6 was deduced by the Lasergene software. Homology analysis was performed using BLASTP (http://blast.ncbi.nlm.nih.gov/Blast.cgi, accessed on 3 August 2021) on the NCBI platform and the sequences were aligned by Clustal W. Conserved domains were located using a conserved domains website (http://www.ncbi.nlm.nih.gov/Structure/cdd/wrpsb.cgi, accessed on 3 August 2021). N-terminal signal peptides were predicted by the SignalP 5.0 Server (http://www.cbs.dtu.dk/services/SignalP/, accessed on 3 August 2021).

### 2.4. Recombinant Protein Expression and Purification

The target sequence was excised from a pGEM-T/AcerOBP6 vector with the specific endonuclease and then cloned into a pET-28a vector. The recombinant plasmid was transformed into BL21 (DE3) *Escherichia coli* cells for protein expression. The recombinant protein expression and purification process were similar to those described by Du et al. [21]. According to the SDS-PAGE analysis, the recombinant proteins were the insoluble inclusion bodies. His-tag was removed by treatment with recombinant thrombin (Sangon). Then, the protein without His-tag was further purified and confirmed by SDS-PAGE. Finally, the BSA method was used to measure the protein concentration.

### 2.5. Fluorescence Competitive Binding Assay

A fluorescence competitive binding assay with 1–NPN (N-phenyl-1-naphthylamine) was carried out on a RF-5301PC (Shimadzu, Japan) fluorescence spectrophotometer using a 1 cm of light path quartz cuvette. Afterwards, the fluorescent probe N-phenyl-1-naphthylamine (1–NPN) was dissolved in methanol to yield a 1 mM stock solution. In order to measure the affinity of 1–NPN with AcerOBP6, a 1 μM solution of the protein in 50 mM of Tris-HCl buffer (pH 7.4) was titrated with aliquots of 1 mM 1–NPN dissolved in methanol of HPLC purity grade to final concentrations of 1–10 μM, before the resulting fluorescence intensities were recorded. The binding constant of 1-NPN to AcerOBP6 (K_1–NPN_) was calculated using Scatchard plots of the binding data in GraphPad Prism 8.0.

Taking 1–NPN as the fluorescent reporter, we measured the binding affinity of each odorant by competitive binding assays using. All the 33 ligands (listed in Table S2) were dissolved in spectrally pure grade methanol. We titrated the odorants in a solution containing AcerOBP6 protein (1 μM) and 1–NPN (1 μM) to the final concentrations of 1–10 μM, respectively. The binding affinity constants (Ki) of the ligands for AcerOBP6 were calculated according to the following equation: Ki = [IC50]/(1 + [1–NPN]/K_1–NPN_), where [IC50] represents the concentration of the competitor reduced to half-maximal fluorescence intensity, [1–NPN] represents the free concentration of 1–NPN, and K_1–NPN_ represents the dissociation constant of the AcerOBP6/1–NPN complex.

### 2.6. dsRNA Synthesis and RNAi Verification

The plasmid extracted in Section 2.3 was used as a template to generate 2 dsRNA fragments of AcerOBP6. Meanwhile, dsRNA was synthesized using a T7 RiboMAX™ Express RNAi System (Promega, Madison, WI, USA); the primers used for dsRNA generating are listed in Table S1. The final concentration of dsRNA generation was diluted to 2.5 μg/μL. The concentration and integrity of dsRNA were determined using NanoDrop and agarose gel electrophoresis (1%). Forager bees were collected and fed with AcerOBP6 dsRNA (8 μg for each bee), and then maintained in an artificial climate incubator (65–75% RH; 28 ± 0.4 °C). At the same time, the GFP dsRNA and 30% sugar were employed as the control groups. Each experiment group included 30 bees, and 3 biological repeats were performed. At times of 24, 48, and 96 h after feeding, the RNAi effects were verified by qRT-PCR.

### 2.7. EAG Assays Based on RNAi

The antennae of forager bees were dissected from the bottom and immediately fixed to both sides of a two-pronged electrode using Spectra 360 electrode gel (Parker Laboratories Inc., Fairfield, NJ, USA). The odorants were dissolved in liquid paraffin to final concentrations of 300 μg/μL, and liquid paraffin was adopted as a blank control. A filter paper strip (3 cm × 1cm) filled with 10 μL tested solution was prepared and put into the tube of Pasteur Pipette, which was connected with the gas feeder equipment. The stimulation time of each odor was 0.5 s and the interval was 30 s. Three technical repetitions were carried out, and ten antennae of female adults were tested for each compound. Finally, the EAGPro software (Syntech) was used to analyze the EAG response recordings.

### 2.8. Statistical Analysis

All statistical tests were conduct using SPSS version 26.0. The normality and homoscedasticity of data were verified using Shapiro-Wilk and Homogeneity of variance test. Data from multiple groups were compared using One-Way ANOVA, followed by Bonferroni multiple comparison test for paired comparison. Data from two groups were compared using Student’s *t*-test. A two-sided *p*-value of 0.05 was considered to be statistically significant.

## 3. Results

### 3.1. Sequence Characteristic of AcerOBP6

**Figure 1:**
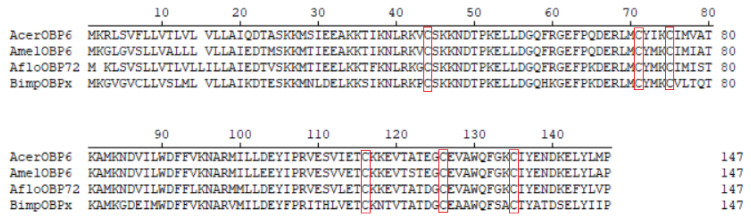
Amino acid sequence alignments of AcerOBP6 with other homologous proteins. Acer, *Apis cerana cerana*; Amel, *Apis mellifera*; Aflo, *Apis florea*; Bimp, *Bombus impatiens*. Six conserved cysteines are boxed with the red frame.

The open reading frame (ORF) of AcerOBP6 was 441 bp, encoding 146 amino acid residues of 16.95 kD. A hydrophobic signal peptide with 24 amino acids was identified and the deduced amino acid sequence had six conserved cysteines, which was a typical motif of classical OBPs (Figure 1).

The results of homology analysis for homologous proteins showed that AcerOBP6 had a high identity with its sibling species (Figure 1), such as 90% to AmelOBP6 and 88% to AfloOBP72. However, it had a relatively low identity with other genera species, such as 69% to BimpOBPx.

### 3.2. Expression Profiles of AcerOBP6

**Figure 2:**
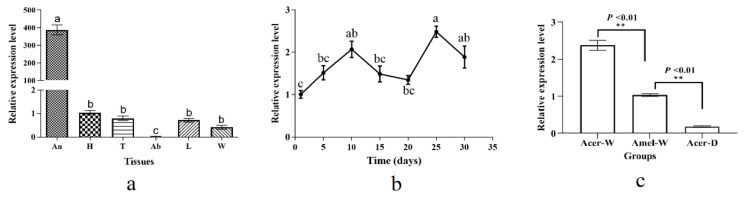
Expression profiles of AcerOBP6. (**a**) Relative expression levels of AcerOBP6 in different tissues (An: antennae; H: head without antennae; T: thorax; Ab: abdomen; L: legs; W: wings) (**b**) in different development stages and (**c**) in different sexes and species. Different lowercase letters above bars indicate significant differences in expression between groups (*p* < 0.05). The error bars represent the mean ± standard error. Double asterisks indicate significant differences between the two means (*p* < 0.01). Acer-W: antennae of worker bees from *A. cerana cerana*; Amel-W: antennae of worker bees from *A. mellifera ligustica*; Acer-D: Antennae of drones from *A. cerana cerana*.

In order to determine the spatio-temporal expression characteristic of AcerOBP6, qRT-PCR was performed. The mRNA transcripts were expressed highly in the antennae and weakly in other tissues (*F* = 15.54, *p* < 0.01), which had the lowest level in the abdomen (Figure 2a). During the developmental stages, AcerOBP6 had a relatively low expression in the newly emerged worker bees, which increased as they grew. Of the adult bees, the expression was shown to be relatively higher in the 25-day-old bees, but the change was not as acute across all the stages (Figure 2b). In addition, we compared the expression of AcerOBP6 between the worker and drone bees, as well as that of AmelOBP6. As revealed by the results, the transcripts were expressed more abundantly in workers than in drones (*t* = 27.97, *p* < 0.01), and the homologous gene was expressed more highly in *A. c. cerana* than in *A. mellifera ligustica* (*t* = 16.62, *p* < 0.01) (Figure 2c).

### 3.3. Ligand Binding Characteristic of AcerOBP6

The recombinant protein of AcerOBP6 was constructed and purified. On this basis, we found that the band size in the SDS-PAGE electrophoresis gel was consistent with the expected molecular weight (Figure S1), and the concentration of the protein was 0.086 mg/mL.

**Figure 3:**
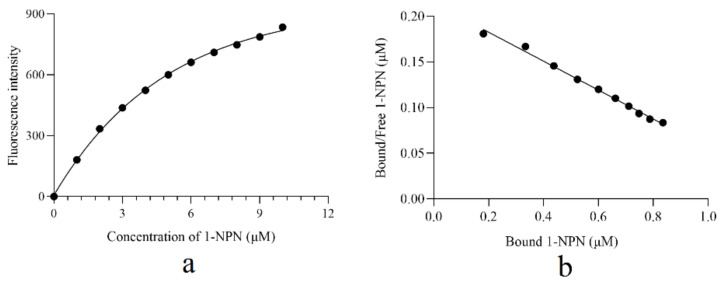
The binding curve of AcerOBP6 with 1-NPN. (**a**) The binding curve of 1-NPN to AcerOBP6. (**b**) The relative Scatchard plot of 1-NPN with AcerOBP6.

First of all, the binding affinity of 1-NPN to AcerOBP6 was tested (Figure 3a). Beyond that, Linear Scatchard plots were obtained (Figure 3b) and a binding constant (Kd) of 6.3 µM was calculated. Then, using 1-NPN as a fluorescent probe, we measured the binding affinities of AcerOBP6 to candidate pheromones and plant volatiles. The values of IC_50_ and the dissociation constants (Ki) were listed in Table S2. The smaller Ki values indicated that there was a stronger affinity between ligand and protein. Among the 34 tested ligands, most of the candidate odors had binding affinities with AcerOBP6. In addition, Linolenic acid, (+)-3-Carene, ethyl-trans-cinnamate, and eugenol exhibited a relatively higher affinity. The Ki values were 1.6, 3.0, 3.7, and 4.7 µM, respectively.

However, there were 11 compounds that could not bind or had a weak binding affinity with AcerOBP6, including seven brood pheromones (methyl oleate, methyl stearate, methyl palmitate, ethyl oleate, ethyl linoleate, ethyl linoleate, and methyl linolenate), four plant volatiles (α-caryophllene, α-farnesene, ethyl cinnamate, and α-ethyl linolenic), and one alarm pheromone (isoamyl acetate).

### 3.4. Further Results from RNAi and EAG Assays

**Figure 4:**
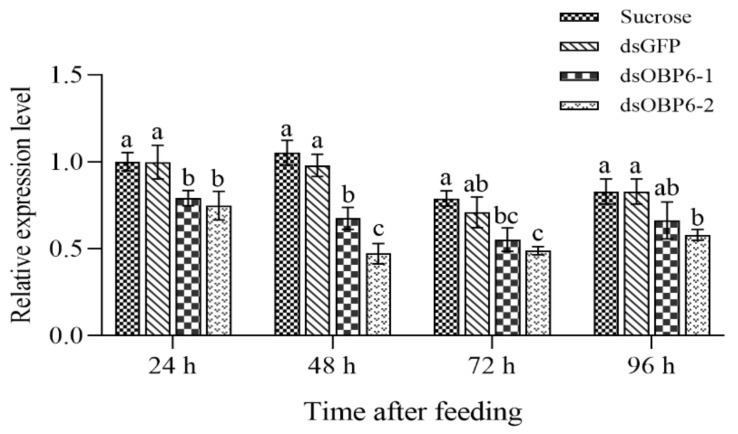
Silencing effect on the mRNA expression levels of AcerOBP6 after feeding worker bees at different times. The error bars represent the mean ± standard error, and means with different lowercase letters indicate significantly between 4 treatment groups of each time (*p* < 0.05).

To further assess the binding specificity of AcerOBP6, an EAG assay based on RNAi was performed. Firstly, we detected the effect of silencing after feeding dsRNA at four different time points. According to qRT-PCR verification results, the transcript levels of the RNAi groups were all significantly lower than those of the control groups (*p* < 0.05). Furthermore, the silencing effect was better at 48 h after feeding (dsOBP6-1 decreased by 35.9%, and dsOBP6-2 reduced by 55.2%) than at 0, 24, or 96 h. In addition, the expression levels of the dsGFP groups did not change significantly compared to the sugar water group (*p* > 0.05) (Figure 4), indicating that the RNAi experiment was successful.

**Figure 5:**
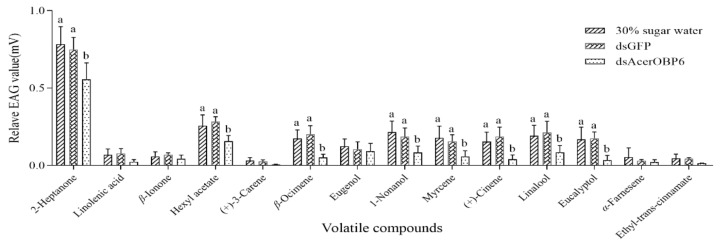
Normalized EAG response of worker bee antennae to selected ligands before and after feeding dsOBP6. The error bars represent the mean ± standard error. Means with different lowercase letters indicate significant differences between three treatment groups of each volatile (*p* < 0.05).

According to the results, we selected the dsOBP6-2 dsRNA to feed to the workers. Then, after 48 h the bees were used in the following EAG assay. A total of 14 ligands with relatively low Ki values were used in the EAG assay. As shown in Figure 5, 2-heptanone triggered a strong EAG response to the worker bee antennae. All the EAG values of the tested ligands were somewhat reduced in the dsOBP6 groups, in which the response to 2-heptanone, hexyl acetate, β-ocimene, 1-nonanol, Myrcene, (+) -cinene, linalool and eucalyptol were significantly decreased (*p* < 0.05). Notably, there was no significant difference in response between the negative control and dsGFP groups. From the results, we also found that the EAG value would not necessarily change greatly after RNAi, although the odors had a strong binding affinity in the fluorescence competitive binding assay.

## 4. Discussion

Insect OBPs are thought to be the pioneer proteins that are involved in the process of odor discrimination, binding, and delivery of chemical stimuli to ORs. In this research, we determined the sequence and expression features and the binding affinity of an OBP gene, AcerOBP6, from the antennae of *A. cerana cerana.* According to the sequence analysis, we observed that AcerOBP6 had a small molecular weight, six highly conserved cysteines, and contained a signal peptide, indicating that AcerOBP6 belongs to a typical ‘classic’ OBP family [23].

The potential biological significance of genes could be derived from their transcript expression profiles [24]. It is widely believed that the OBPs specifically expressed in antennae play a role in the olfaction of insects [25,26], while the ones expressed elsewhere may get involved in the non-chemosensory processes [27,28,29]. According to the qRT-PCR results, the AcerOBP6 transcript was primarily expressed in the antennae of forager bees and significantly more expressed than in the antennae of drones, suggesting that AcerOBP6 may play an essential role in recognizing general odorants compared to sex pheromones. Apart from that, AmelOBP6 was also expressed exclusively in the antennae of forager bees [9]. It was noteworthy that the expression value of AcerOBP6 mRNA was significantly higher than AmelOBP6, indicating that the olfactory capabilities and the importance of homologous olfactory proteins may be different in the sibling species.

Competitive fluorescence binding assays have been widely used in the binding affinity studies of insect OBPs [30]. In all 33 candidate volatiles, AcerOBP6 had been found to possess a relatively strong binding capability to 20 ones, with Kd values <10 µmol/L, including the queen pheromone (9-ODA), one alarm pheromone (2-Heptanone), two Nasonov pheromones (Geranionl and Farnesol) and most of the floral volatiles. In other words, AcerOBP6 was a broad-spectrum binding protein, which had similar binding properties to those of other insect OBPs, such as AipsGOBP1 and AlepGOBP2 [31,32]. At the same time, several studies had reported OBP functions in *A. cerana cerana* including AcerOBP1, AcerOBP10, AcerOBP11, and AcerOBP15, in which AcerOBP1 and AcerOBP15 were the proteins that were mainly involved in pheromone binding [19,21,33,34]. In addition, this binding characteristic of AcerOBP6 was also consistent with the structure of the PBP-GOBP domain. These results indicated that one OBP gene may regulate the recognition process for various of odors [35]. Notably, linolenic acid showed a higher binding affinity among these ligands (Ki = 1.67 µM). As is well known, Linolenic acid is one of the major fatty acids of the omega-3 family, which plays an important role in the growth and normal metabolism of the body. It cannot be synthesized in vivo and should be obtained from food. However, linolenic acid and linoleic acid exist in some floral pollen [36,37]. It was speculated that AcerOBP6 could assist bees to collect pollen by identifying linolenic acid from flowers, which provide a nutritional supply for larvae growth and ensure the stability of the colony. Except for linolenic acid, AcerOBP6 could also bind with other plant volatiles strongly, such as 3-carene and ethyl-trans-cinnamate. Studies have reported that 3-carene is the main component of monoterpenes in plant volatile organic compounds and plays a potential role in attracting parasitic wasps [38,39]. Ethyl *trans*-cinnamate is isolated from lodgepole pine, *Pinus contorta*, which has been bioassayed as an antifeedant for pine weevils [40] but it is also described to have a sweet honey odor [41]. Therefore, the strong affinity of AcerOBP6 with plant volatiles suggested that AcerOBP6 mainly participated in pollen and nectar foraging in *A. cerana cerana*. Meanwhile, AcerOBP6 had a very weak binding affinity with brood pheromones. This might be because these ester pheromones are the non-volatile components, while insect OBPs mainly bind with volatile molecules [42].

In order to further confirm whether AcerOBP6 participates in binding of those volatiles screened in the competitive fluorescence binding assay, RNAi combined with EAG methods were performed. Similar to other previous research, we used the feeding method to accomplish RNAi [21,43,44]. According to the qRT-PCR results, the gene was successfully silenced. The EAG recording values of the tested volatiles were all decreased in varying degrees compared with the control groups. Contrary to our expectation, the three odors which had high binding affinities with AcerOBP6 failed to elicit a strong response to the antennae and were not changed significantly in the EAG assay. To our knowledge, the olfactory properties of honeybees are very sensitive, but they have a small number of OBPs compared to other insects [9]. It was possible that each OBP of the honeybee has an extensive binding capacity with odorant molecules. Our results indicate that AcerOBP6 could bind with odors such as linolenic acid, 3-carene, and ethyl-trans-cinnamate. At the same time, these odors might also be bound by other OBPs. The previous research about the discrimination to oviposition deterrent volatile in *Bemisia tabaci* also reported the similar viewpoint [45].

In addition, 2-heptanone elicited strong EAG responses to workers’ antennae and displayed a significant change after the bees dealt with dsRNA. In other words, *A. cerana cerana* was sensitive to 2-heptanone and the chemical compound was likely the specific binding ligand of AcerOBP6. 2-Heptanone is an alarm pheromone that delivers dangerous signals in the colony to trigger the defensive response of bees [46]. What’s more, it is found that 2-heptanone could also function as a chemical marker on flowers that have been visited by other bees, thus improving the foraging efficiency of foragers [47,48].

## 5. Conclusions

To conclude, an *E. coli* expression system was developed to obtain an odorant binding protein of *A. cerana cerana*. The amino acid sequence of this protein was highly homologous with AmelOBP6. AcerOBP6 was mainly expressed in the antennae of forager bees. This protein showed a good affinity with most floral volatiles and some bee pheromones, except for the larval pheromones. Linolenic acid was the strongest binding odorants among the selected compounds, which is an odorant compound in some floral pollen. Using the combined methods of EAG and RNAi assays, we further investigated the special ligands of AcerOBP6 including 2-heptanone, hexyl acetate, eugenol, β-ocimene, 1-nonanol, myrcene, (+) -cinene, and linalool. The findings of this study suggested that AcerOBP6 plays an important role in the odorant binding process, especially in modulating pollen foraging behavior of workers.

## Data Availability

Data are contained within the article or supplementary material.

## Supplementary Materials

The following are available online at https://www.mdpi.com/article/10.3390/insects12110955/s1. Figure S1: The SDS-PAGE gel electrophoresis result; Table S1: Primers used in the experiment; Table S2: Competitive binding affinity of candidate ligands with AcerOBP6.

## References

[1] Ache B.W., Young J.M. (2005). Olfaction: Diverse species, conserved principles. Neuron.

[2] Sun X., Wang M.Q., Zhang G. (2011). Ultrastructural observations on antennal sensilla of *Cnaphalocrocis medinalis* (Lepidoptera: Pyralidae). Microsc. Res. Tech..

[3] Mo J.C., Wang C.P., Wei J.Q. (2019). Advance in the research on insect peripheral olfactory system. Acta Agric. Univ. Jiangxiensis.

[4] Leal W.S. (2013). Odorant reception in insects: Roles of receptors, binding proteins, and degrading enzymes. Annu. Rev. Entomol..

[5] Brito N.F., Moreira M.F., Melo A.C. (2016). A look inside odorant-binding proteins in insect chemoreception. J. Insect Physiol..

[6] Ban L.P., Scaloni A., D’Ambrosio C., Zhang L., Yan Y.H., Pelosi P. (2003). Biochemical characterization and bacterial expression of an odorant-binding protein from *Locusta migratoria*. Cell. Mol. Life Sci..

[7] Yang M.X., Tan K., Radloff S.E., Phiancharoen M., Hepburn H.R. (2010). Comb construction in mixed-species colonies of honeybees, *Apis cerana* and *Apis mellifera*. J. Exp. Biol..

[8] Diao Q.Y., Sun L.X., Zheng H.J., Zeng Z.J., Wang S.Y., Xu S.F., Zheng H.Q., Chen Y.P., Shi Y.Y., Wang Y.Z. (2018). Genomic and transcriptomic analysis of the Asian honeybee *Apis cerana* provides novel insights into honeybee biology. Sci. Rep..

[9] Forêt S., Maleszka R. (2006). Function and evolution of a gene family encoding odorant binding-like proteins in a social insect, the honey bee (*Apis mellifera*). Genome Res..

[10] Zhao H., Du Y., Gao P., Wang S., Pan J., Jiang Y. (2016). Antennal transcriptome and differential expression analysis of five chemosensory gene families from the Asian honeybee *Apis cerana cerana*. PLoS ONE.

[11] Danty E., Briand L., Michard-Vanhee C., Perez V., Arnold G., Gaudemer O., Huet D., Huet J., Ouali C., Masson C. (1999). Cloning and expression of a queen pheromone-binding protein in the honeybee: An olfactory-specific, developmentally regulated protein. J. Neurosci..

[12] Pesenti M.E., Spinelli S., Bezirard V., Briand L., Pernollet J.C., Tegoni M., Cambillau C. (2008). Structural basis of the honeybee PBP pheromone and pH-induced conformational change. J. Mol. Biol..

[13] Li H.L., Gao Q.K., Cheng J.A. (2008). Cloning and spatio-temporal expression of cDNA encoding pheromone binding protein ASPl in Apis cerana cerana (Hymenoptera Apidae). Acta Entomol. Sin..

[14] Weng C., Fu Y., Jiang H., Zhuang S., Li H. (2015). Binding interaction between a queen pheromone component HOB and pheromone binding protein ASP1 of *Apis cerana*. Int. J. Biol. Macromol..

[15] Danty E., Michard Vanhee C., Huet J.C., Genecque E., Pernollet J.C., Masson C. (1997). Biochemical characterization, molecular cloning and localization of a putative odorant-binding protein in the honeybee *Apis mellifera* L. (Hymenoptem Apidea). FEBS. Lett..

[16] Briand L., Nespoulous C., Huet J.C., Takahashi M., Pernollet J.C. (2001). Ligand binding and physico-chemical properties of ASP2, a recombinant odorant-binding protein from honeybee (*Apis mellifera* L.). Eur. J. Biochem..

[17] Li H.L., Song X.M., Wu F., Qiu Y.L., Fu X.B., Zhang L.Y., Tan J. (2020). Chemical structure of semiochemicals and key binding sites together determine the olfactory functional modes of odorant-binding protein 2 in Eastern honeybee, *Apis cerana*. Int. J. Biol. Macromol..

[18] Iovinella I., Dani F.R., Niccolini A., Sagona S., Michelucci E., Gazzano A., Turillazzi S., Felicioli A., Pelosi P. (2011). Differential expression of odorant-binding proteins in the mandibular glands of the honeybee according to caste and age. J. Proteome Res..

[19] Song X.M., Zhang L.Y., Fu X.B., Wu F., Tan J., Li H.L. (2018). Various Bee Pheromones Binding Affinity, Exclusive Chemosensillar Localization, and Key Amino Acid Sites Reveal the Distinctive Characteristics of Odorant-Binding Protein 11 in the Eastern Honeybee, *Apis cerana*. Front. Physiol..

[20] Guo D.Z., Hao C.H., Cui X.P., Wang Y., Liu Z.G., Xu B.H., Guo X.Q. (2021). Molecular and functional characterization of the novel odorant-binding protein gene AccOBP10 from *Apis cerana cerana*. J. Biochem..

[21] Du Y.L., Xu K., Zhao H.T., Jiang Y.S., Li H.Q. (2021). Identification and functional characterization of AcerOBP15 from *Apis cerana cerana* (Hymenoptera: Apidae). Apidologie.

[22] Livak K.J., Schmittgen T.D. (2001). Analysis of relative gene expression data using real-time quantitative PCR and the 2^−ΔΔCt^ method. Methods.

[23] Zhou J.J., Vieira F.G., He X.L., Smadja C., Liu R., Rozas J., Field L.M. (2010). Genome annotation and comparative analyses of the odorant-binding proteins and chemosensory proteins in the pea aphid *Acyrthosiphon pisum*. Insect Mol. Biol..

[24] Hull J.J., Peretra O.P., Snodgrass L. (2014). Cloning and expression profiling of odorant-binding proteins in the tarnished plant bug, *Lygus lineolaris*. Insect Mol. Biol..

[25] Pelosi P., Iovinella I., Felicioli A., Dani F.R. (2014). Soluble proteins of chemical communication: An overview across arthropods. Front. Physiol..

[26] Iovinella I., Cappa F., Cini A., Petrocelli I., Cervo R., Turillazzi S., Dani F.R. (2018). Antennal protein profile in honeybees: Caste and task matter more than age. Front. Physiol..

[27] Ishida Y., Ishibashi J., Leal W.S. (2013). Fatty acid solubilizer from the oral disk of the blowfly. PLoS ONE.

[28] William B.W., Amit R., Peter A., Fredrik S., Bill S.H., Mattias C.L. (2019). Transcriptome Analysis of Gene Families Involved in Chemosensory Function in *Spodoptera littoralis* (Lepidoptera: Noctuidae). BMC Genom..

[29] Pelosi P., Iovinella I., Zhu J., Wang G., Dani F.R. (2018). Beyond chemoreception: Diverse tasks of soluble olfactory proteins in insects. Biol. Rev. Camb. Philos. Soc..

[30] Pelosi P., Mastrogiacomo R., Iovinella I., Tuccori E., Persaud K.C. (2014). Structure and biotechnological applications of odorant binding proteins. Appl. Microbiol. Biot..

[31] Huang G.Z., Liu J.T., Zhou J.J., Wang Q., Dong J.Z., Zhang Y.J., Li X.C., Li J., Gu S.H. (2018). Expressional and functional comparisons of two general odorant binding proteins in *Agrotis ipsilon*. Insect Biochem. Mol..

[32] Zhang X.Q., Yan Q., Li L.L., Xu J.W., Mang D., Wang X.L., Hoh H.H., Ye J., Ju Q., Ma Y. (2020). Different binding properties of two general-odorant binding proteins in *Athetis lepigone* with sex pheromones, host plant volatiles and insecticides. Pestic. Biochem. Physiol..

[33] Weng C., Zhang L.Y., Zhao L., Fu Y.X., Luo C., Li H.L. (2013). Prokaryotic expression and ligand binding characteristics of pheromone binding protein ASP1 in the Chinses honeybee (*Apis cerana cerana*). Acta Entomol. Sin..

[34] Wu F., Huang J.J., Tan J., Tang M.Z., Li H.L. (2016). Molecular cloning, prokaryotic expression and lignand-binding characterization of a novel pheromone binding protein OBP10 in *Apis cerana cerana* (*Hymenoptera*: *Apidae*). Acta Entomol. Sin..

[35] Sun S.F., Zeng F.F., Yi S.C., Wang M.Q. (2019). Molecular screening of behaviorally active compounds with CmedOBP14 from the rice leaf folder *Cnaphalocrocis medinalis*. J. Chem. Ecol..

[36] Manning R. (2001). Fatty acids in pollen: A review of their importance for honeybees. Bee World.

[37] Arien Y., Dag A., Shafir S. (2018). Omega-6:3 Ratio More Than Absolute Lipid Level in Diet Affects Associative Learning in Honeybees. Front. Psychol..

[38] Adal A.M., Sarker L.S., Lemke A.D., Mahmoud S.S. (2017). Isolation and functional characterization of a methyl jasmonate-responsive 3-Carene synthase from Lavandula x intermedia. Plant Mol. Biol..

[39] Van Dam N.M., Qiu B.-L., Hordijk C.A., Vet L.E.M., Jansen J.J. (2010). Identification of Biologically Relevant Compounds in Aboveground and Belowground Induced Volatile Blends. J. Chem. Ecol..

[40] Bratt K., Sunnerheim K., Nordenhem H., Nordlander G., Langström B. (2001). Pine weevil (*Hylobius abietis*) antifeedants from lodgepole pine (*Pinus contorta*). J. Chem. Ecol..

[41] Aceña L., Vera L., Guasch J., Busto O., Mestres M. (2011). Chemical characterization of commercial Sherry vinegar aroma by headspace solid-phase microextraction and gas chromatography-olfactometry. J. Agric. Food. Chem..

[42] Vogt R.G., Prestwich G.D., Lerner M.R. (1991). Odorant-binding-protein subfamilies associate with distinct classes of olfactory receptor neurons in insects. J. Neurobiol..

[43] Swevers L., Huvenne H., Menschaert G., Kontogiannatos D., Kourti A., Pauchet Y., Ffrench-Constant R., Smagghe G. (2013). Colorado potato beetle (Coleoptera) gut transcriptome analysis: Expression of RNA interference-related genes. Insect Mol. Biol..

[44] San Miguel K., Scott J.G. (2016). The next generation of insecticides: dsRNA is stable as a foliar-applied insecticide. Pest. Manag. Sci..

[45] Li F.Q., Li D., Dewer Y., Qu C., Yang Z., Tian J.H., Luo C. (2019). Discrimination of oviposition deterrent volatile β-ionone by odorant-binding proteins 1 and 4 in the whitefly *Bemisia tabaci*. Biomolecules.

[46] Shearer D.A., Boch R. (1965). 2-heptanone in the mandibular gland secretion of the honeybee. Nature.

[47] Reith J.P., Wilson W.T., Levin M.D. (1986). Repelling honey bees from insecticide treated flowers with 2-heptanone. J. Apicult. Res..

[48] Vallet A., Cassier P., Lensky Y. (1991). Ontogeny of the fine structure of the mandibular glands of the honeybee (*Apis mellifera* L.) workers and the pheromonal activity of 2-heptanone. J. Insect Physiol..

