# Supplementary material for: Expression Profile and Ligand Screening of a Putative Odorant-Binding Protein, AcerOBP6, from the Asian Honeybee
Source: Insects. 2021 Oct 20;12(11):955. doi: 10.3390/insects12110955 (PMC8622152; doi:10.3390/insects12110955)
Supplement: Supplementary file 1 [file insects-12-00955-s001.zip › Figure S1.pdf]

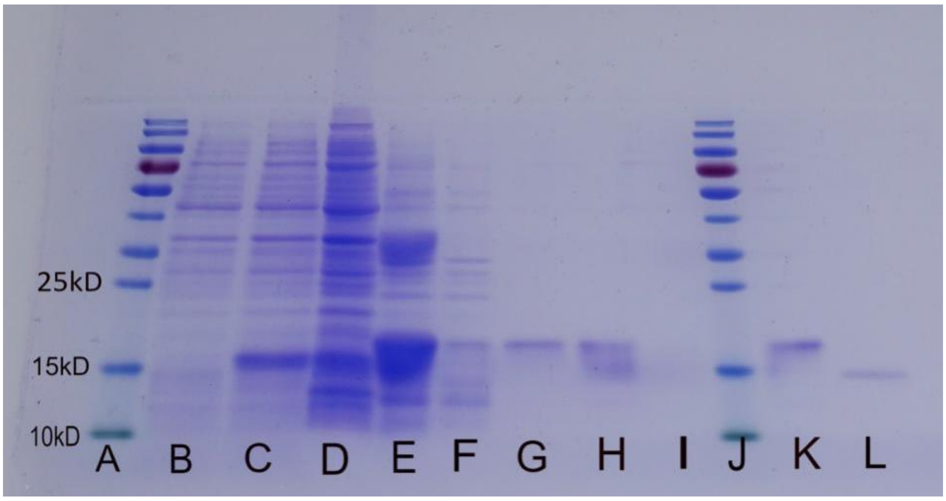

Figure S1. The SDS-PAGE gel electrophoresis result. A and J, Protein molecular weight marker; B, Bacterial solution before IPTG induction; C, Bacterial solution after IPTG induction; D, Centrifugal supernatant after ultrasonication; E, Centrifugal precipitation after ultrasonication; F. Protein waste liquid; G-I, AcerOBP6 eluted with 50, 100 and 300 mmol/L imidazole solution, respectively; K,. AcerOBP6 after renaturation and concentration; L, Purified AcerOBP6 protein after removal of his-tag.
