# Supplementary material for: Expression Profile and Ligand Screening of a Putative Odorant-Binding Protein, AcerOBP6, from the Asian Honeybee
Source: Insects. 2021 Oct 20;12(11):955. doi: 10.3390/insects12110955 (PMC8622152; doi:10.3390/insects12110955)
Supplement: Supplementary file 1 [file insects-12-00955-s001.zip › Table S1.pdf]

Table S1. Primers used in the experiment.

Primers	Sequences (5'-3')
For qRT-PCR	
OBP6-F	TCTCCCTGCTGGTTGCGTT
OBP6-R	GCTCATGGTATCCTCGATCGCT
Arp1-F	ACTACGGCCGAACGTGAAAT
Arp1-R	GGAAAAGAGCCTCGGGACAA
For recombinant expression	
OBP6-F	cgcggatccAAAAAGATGAGCATCGAGG
OBP6-R	cccaagcttTCATGGCATTAAATAGAGCTC
For dsRNA synthesis	
OBP6-F1	ACGGTCAATTCAGAGGCGAA
OBP6-R1	CTCCGTAGCTGTCACTTCCT
T7-OBP6-F1	taatacgactcactatagggACGGTCAATTCAGAGGCGAA
T7-OBP6-R1	taatacgactcactatagggCTCCGTAGCTGTCACTTCCT
OBP6-F2	AGCATCGAGGAAGCGAAGAA
OBP6-R2	ACATTAGCCTCTCGTCCTGC
T7-OBP6-F2	taatacgactcactatagggAGCATCGAGGAAGCGAAGAA
T7-OBP6-R2	taatacgactcactatagggACATTAGCCTCTCGTCCTGC
GFP-F	CACAAGTTCAGCGTGTCC
GFP-R	CTGGGTGCTCAGGTAGTG
T7-GFP-F	taatacgactcactatagggCACAAGTTCAGCGTGTCC
T7-GFP-R	taatacgactcactatagggCTGGGTGCTCAGGTAGTG

The wavy line represents the restriction site. The underline represents the T7 promote sequence.
