# Supplementary material for: Expression Profile and Ligand Screening of a Putative Odorant-Binding Protein, AcerOBP6, from the Asian Honeybee
Source: Insects. 2021 Oct 20;12(11):955. doi: 10.3390/insects12110955 (PMC8622152; doi:10.3390/insects12110955)
Supplement: Supplementary file 1 [file insects-12-00955-s001.zip › Table S2.pdf]

Table S2. Competitive binding affinity of candidate ligands with AcerOBP6.

Category	Ligands	IC50（μM）	Ki (μM)
Queen pheromone			
	9-ODA 9-Oxo-(E)-2-decenoic acid	7.55	7.18
Alarm pheromone			
	2-Heptanone	6.15	5.85
	Isoamyl acetate	-	-
Nasonov pheromone			
	Geraniol	7.46	7.09
	Nerol	-	-
	Farnesol	5.56	5.29
Brood pheromone			
	Methyl oleate	-	-
	Methyl stearate	-	-
	Methyl palmitate	-	-
	Ethyl oleate	-	-
	Ethyl linoleate	-	-
	Methyl linoleate	-	-
	Methyl linolenate	-	-
Plant volatiles			
	1- Nonanol	5.56	5.29
	1-Octen-3-ol	7.59	7.22
	Myrcene	5.42	5.16
	β-Ionone	5.45	5.18
	β-Caryophllene	-	-
	Linolenic acid	1.76	1.67
	Ethyl acetate	5.56	5.28
	Eucalyptol	5.88	5.59
	Eugenol	4.90	4.66
	Ethyl-trans-cinnamate	3.93	3.74
	(+) -3-Carene	3.17	3.02
	β-Ocimene	5.54	5.27
	Nonanal	8.91	8.48
	α-Farnesene	-	-
	Ethyl cinnamate	-	-
	α-Ethyl linolenic	-	-
	Linalool	5.68	5.41
	(+) -Cinene	5.53	5.26
	β-Pinene	6.67	6.35
	Methyl salicylate	10.07	9.58

IC50: the concentration of ligand compounds binding with 50% 1-NPN; Ki: dissociation constant. Short dash: it was not possible to calculate the IC50 value.
